# Anti-Inflammatory Activity and Mechanism of Cryptochlorogenic Acid from *Ageratina adenophora*

**DOI:** 10.3390/nu14030439

**Published:** 2022-01-19

**Authors:** Xiaoping Ma, Samuel Kumi Okyere, Liwen Hu, Juan Wen, Zhihua Ren, Junliang Deng, Yanchun Hu

**Affiliations:** 1 Key Laboratory of Animal Diseases and Environmental Hazards of Sichuan Province, College of Veterinary Medicine, Sichuan Agricultural University, Chengdu 611130, China; mxp886@sicau.edu.cn (X.M.); samuel20okyere@gmail.com (S.K.O.); huliwen197@163.com (L.H.); juanwen881010@163.com (J.W.); zhihua_ren@126.com (Z.R.); dengjl213@126.com (J.D.); 2 Key Laboratory of Animal Disease and Human Health of Sichuan Province, Sichuan Agricultural University, Chengdu 611130, China

**Keywords:** *Ageratina adenophora*, cryptochlorogenic acid, anti-inflammatory, nuclear factor-kappa B pathway

## Abstract

*Ageratina adenophora* is an invasive plant known for its toxicity to livestock. Current research on this plant has shifted from toxicity prevention to the beneficial utilization of plant resources. This study was performed to investigate the effects and mechanisms of cryptochlorogenic acid (CCGA) isolated from *Ageratina adenophora* on the inflammatory responses induced by lipopolysaccharide (LPS) in RAW264.7 cells. RAW264.7 cells were pretreated with CCGA (200, 100, and 50 μg/mL) and subsequently stimulated with LPS (1 μg/mL) for 16 h. The cytotoxicity of CCGA was tested using the Cell Counting Kit (CCK8). The mechanism of action of CCGA in attenuating inflammation was also identified using enzyme-linked immunosorbent assay (ELISA), quantitative reverse transcription-polymerase chain reaction, and Western blot. The results showed that CCGA had a maximal safe concentration of 200 mg/mL. Moreover, CCGA reduced the level of nitric oxide (NO) and iNOS in LPS-induced RAW264.7 cells (*p* < 0.01). In addition, CCGA reduced the levels of pro-inflammatory cytokines (TNF-α, IL-1β, IL-6, and IL-8) and cyclooxygenase-2 (COX-2) in LPS-induced RAW264.7 cells at both the mRNA and protein levels (*p* < 0.01). CCGA prevented the activation of nuclear factor-kappa B (NF-kB) in LPS-induced RAW264.7 cells via the inhibition of IKK and IκB phosphorylation and the degradation of IκB proteins (*p* < 0.01). This finding indicated that CCGA isolated from *A. adenophora* may be a potential candidate for the treatment of inflammation-related diseases.

## 1. Introduction

The chief tool used by most organisms against infection is inducing inflammatory responses [1]. However, elevated levels of inflammatory cytokines in the body circulation or in local inflammatory sites result in severe diseases [2,3,4]. Chronic inflammation is linked with various diseases, such as diabetes, depression, and cancer [5,6,7,8,9,10,11,12,13]. Among the inflammatory inducing agents, lipopolysaccharide (LPS) is reported to enhance the production of pro-inflammatory cytokines in macrophages, fibroblasts, and monocytes [14]. Furthermore, it has been reported that LPS-induced inflammatory reactions can occur via the NF-κB signaling pathway [15]. TNF-α cytokine actively takes part in systemic inflammation as well as immune cell regulation [16]. IL-1β is known for activating the plasmalemmal IL-1R1 receptor in various types of cells [17,18]. IL-6 is involved in numerous cellular activities, including cell proliferation, differentiation, and apoptosis [19]. IL-8 is a pro-inflammatory cytokine that is linked to tumor growth and progression [20]. Cyclooxygenase (COX) is an oxidoreductase enzyme noted for the conversion of arachidonic acid to prostaglandins, thereby initiating inflammatory reactions [21]. COX-2 is associated with PGE2 synthesis in numerous examples of inflammation [22]. 

Nitrite oxide (NO) is an inflammatory mediator of the nitric oxide synthase (NOS) family [23]. Three types of NOSs have been identified, namely, inducible NOS (iNOS), neuronal NOS (nNOS), and endothelial NOS (eNOS) [24]. Both nNOS and eNOS are constitutive NOSs, whereas iNOS is activated by cytokines to yield NO [25]. An increase in NO concentration in cells causes cytotoxicity and induces various inflammatory disorders [26,27]. 

Inflammation is linked with the NF-κB pathway. NF-κB is a nuclear transcriptional factor that is linked to both pro- and anti-inflammatory activities [28]. In dormant cells, the NF-κB dimers are joined by inhibitory molecules, such as IκB, to keep them stable in the cytoplasm. LPS activates IKK to initiate the phosphorylation of IκB in cells, which causes the degradation of IκBα protein and the activation of NF-κB translocation into the nucleus to bind target DNAs [29]. 

**Figure 1:**
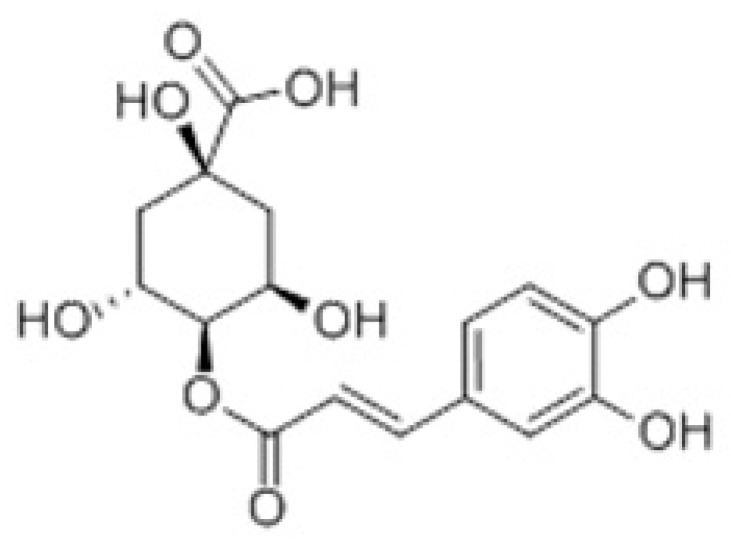
Chemical structure of cryptochlorogenic acid (CCGA).

Inflammation is one of the key drivers of pathogenesis in many diseases; therefore, over the past years, numerous studies have identified and isolated many anti-inflammatory compounds from natural products, especially from plant sources, because they produce fewer side effects compared with commercial drugs. Among these compounds from natural products, polyphenolic compounds in plants are the most widely known to possess anti-inflammation activities [30], whereas phenolic acids are the most abundant among plant polyphenolic compounds. They have been isolated from different plant parts, such as the leaves and seeds of vegetables and the skins of fruits [31]. Among the phenolic acid group, chlorogenic acid is the most well-known and abundant compound known to possess beneficial biological properties, such as anticancer, anti-inflammatory, antioxidant, antimicrobial, and antiviral effects [32,33,34,35]. Cryptochlorogenic acid (CCGA) (Figure 1), a distinct isomer of chlorogenic acid with similar structural features, has also demonstrated potential anti-inflammatory activities in previous studies [36,37].

*Ageratina adenophora* (Spreng.) R. M. King et H. Rob. is one of the most harmful invasive plants in China [38,39,40,41]. It has strong environmental adaptability and breeding ability, which can cause serious ecological disasters and affect the growth of the animal husbandry industry [42]. However, at present, the focus of research on *A. adenophora* has shifted from toxicity prevention to the biological utilization of the plant resource [43,44,45]; thus, researchers have identified several secondary metabolites with pharmacological properties, such as antibacterial, antitumor, anti-obesity, anticancer, and anti-inflammation effects, in this plant resource [46]. Cryptochlorogenic acid (CCA) is one of the major polyphenolic bioactive compounds isolated from *A. adenophora* [46]; however, its effects and mechanism in inflammation are still unknown. Therefore, this work was performed to explore the anti-inflammatory activity of CCGA isolated from *A. adenophora* in order to discover the pharmacological effects of CCGA as well as its molecular pathway.

## 2. Materials and Methods

### 2.1. Plant Sample, Chemicals, and Reagents

The collection, location, and identification of *Ageratina adenophora* for this study were reported in a study by Okyere et al. (2022). Cells: The RAW264.7 murine cell line was purchased from the Wuhan China Typical Culture Preservation Center (Wuhan, China), which offered Short Tandem Repeat (STR) analysis to confirm the authenticity of all cell lines. Lipopolysaccharides (LPS) were derived from *Escherichia coli* O111:4. High-glucose DMEM and CCK-8 were obtained from HyClone. The imported test kit for fetal bovine serum (GIBCO), the nitric oxide (NO) box, and ELISA kits for TNF-α, IL-1 β, IL-6, IL-8, IL-10, and COX-2 were purchased from Nanjing Jiancheng Company Ltd., Nanjing, China. The total RNA extraction kit and fluorescent quantitative PCR kit were obtained from the Beyotime Institute of Biotechnology (Beijing, China). Secondary antibodies were bought from the Beyotime Institute of Biotechnology (Beijing, China). All experiments were undertaken following the approved guidelines and experimental protocols by the Animal Care and Use Committee of Sichuan Agricultural University, China (no. 2019603005).

### 2.2. Extraction of CCGA

#### Preparation of Aqueous Extract from *Ageratina adenophora*

Fifty grams (50) of milled leaves was mixed in 100 mL of water and soaked for 12 h. The upper layer containing the organic solvent was collected and filtered by suction. After rotating evaporation at 105 kpa and 45 °C, the upper layer containing the organic solvent was dissolved in 1000 mL of extraction solution to begin the extraction process. The liquid ratio used for the extraction was methanol: ethyl acetate: water = 2:5:8 (*v/v/v*). After that, the lower part of the aqueous phase was collected and evaporated at 75 °C to obtain a yellowish-brown aqueous extract.

### 2.3. Separation of Cryptochlorogenic Acid by Macroporous Resin

#### 2.3.1. Macroporous Resin Pretreatment

HPD100 macroporous resin was soaked with 95% ethanol for 24 h. After full swelling, it was washed with ethanol and an appropriate amount of water until the effluent had no white turbidity. Afterward, it was soaked in distilled water until there was no smell of alcohol.

#### 2.3.2. Sample Loading and Elution System

The aqueous extract was dissolved in 95% ethanol, and the ratio of ethanol volume to extract was 10:1 (*v/w*). Using 5%, 15%, 65%, and 95% as eluent, the elution amount was 6 times of column volume. The eluent was then collected from below the column. The collected eluent and cryptochlorogenic acid standard were developed using the mixture of methanol and ethyl acetate in a ratio of 95:5 (*v/v*) as the layer-developing agent, and 2% ferric chloride solution was used as the color developing agent. The samples were collected according to the color-developing points for silica gel column chromatography.

### 2.4. Purification of Cryptochlorogenic Acid by Silica Gel Column Chromatography

Step 2.3 was repeated 5–6 times, and the eluate containing chlorogenic acid was combined for the sample of silica gel column chromatography. The silica gel powder was mixed with ethyl acetate and poured into a clean silica gel column. Twice the column volume of ethyl acetate was used as the eluent to stabilize the column, and the bubble-free column was used as the separation column. Samples were dissolved in ethyl acetate in a ratio of 4:1, and 20 mL of the sample was taken and slowly added to the silica gel column of the above cabinet. After loading, the valve was opened to allow the sample to enter the silica gel column. Using an ethyl acetate to methanol ratio of 98:2 (*v*/*v*) and a methanol to ethyl acetate ratio of 95:5 (*v*/*v*) as eluent, the elution amounts were 3 times the column volume and 6 times the column volume, respectively. The eluent containing cryptochlorogenic acid was collected and combined by thin layer chromatography for secondary column passing.

### 2.5. Identification of Monomer Purity by Ultra High Performance Liquid Chromatography

#### 2.5.1. Chromatographic Conditions

In this analysis, an Agilent 1290 ultra-high liquid chromatograph (chromatographic column: XDB-C18(2.1 × 100 mm, 1.8 μm) was used to analyze chlorogenic acid using water (A) and methanol (B) as mobile phases, methanol–0.1% formic acid solution (30:70), a flow rate of 0.3 mL/min, a detection wavelength of 325 nm, and a column temperature of 28 °C. The sample injection volume was 1 μL, and the peak time was between 1.28 min and 12 h.

#### 2.5.2. Making the Standard Curve

The standard chlorogenic acid of 0.7 mg was accurately weighed and poured into a 3.5 mL volumetric flask; after that, it was dissolved in methanol, ultrasonic, and cooled. After cooling, 0.2 mg/mL concentration solution was prepared using methanol. The prepared solution was then diluted to different concentrations (0.01, 0.05, 0.10, 0.15, and 0.20 mg/mL), and 1 μL of the above standard solution and sample in sequence were loaded. The peak area was determined by ultra-high performance liquid chromatography (UHPLC) according to the chromatographic conditions in 2.5.1. The standard curve was drawn with the peak area as the ordinate and the standard concentration as the abscissa.

#### 2.5.3. Determination of Cryptochlorogenic Acid in *Ageratina adenophora*

An amount of 0.7 mg of the extract monomer was weighed and fixed into 3.5 mL of chromatographic methanol. UHPLC determination was conducted according to the chromatographic conditions in 2.5.1, and the content of cryptochlorogenic acid in the extracted monomer was calculated according to the standard curve.

### 2.6. Cell Viability Using CCK8 Method

The cytotoxicity of CCGA was investigated using the CCK-8 (cell counting kit8) assay. RAW264.7 cells in the logarithmic growth phase were used for this experiment. After counting with a cell counting plate, 100 μL of cell suspension with 5 × 10^4^ cells was seeded on a 96 well plate and cultured at 37 °C and 5% CO_2_ for 24 h. After 24 h of culture at 37 °C and 5% CO_2_, CCGA was released at 400, 200, 100, and 50 μg/mL concentrations for 24 h in DMEM. DMEM was added to the 96-well plate, blank group, and LPS group. After 4 h of continuous culture, 100 μL of cells without stimulation DMEM was added to the blank control group. The LPS group and experimental group were induced with LPS solution at a concentration of 1 μg/mL. for 16 h. After the replacement of fresh DMEM, 10 μL of CCK-8 reagent was added to each well and incubated at 37 °C for 4 h. Each group was set with 3 multiple wells and blank wells (with DMEM and CCK-8 solution, without cells). The absorbance was measured at a 450 nm wavelength. The data are represented as means ± standard deviation. Cell viability was calculated as:


Cell viability (%) = (treatment well OD-blank well OD) /(blank group OD-blank well OD)×100%

From the experimental results of CCK-8, the concentration groups of 200, 100, and 50 μg/mL CCGA were selected for further experiments. Therefore, we designed an experimental study of a blank control group, LPS group, and experimental group with concentrations of 200, 100, and 50 μg/mL. Each group was provided with 3 multiple wells. The experiment was performed in triplicate.

### 2.7. Measurement of Nitric Oxide in RAW264.7 Cells

Nitric oxide levels were measured following the procedure by Ying et al. [25]. RAW264.7 cells were pretreated with CCGA at 50–200 μg/mL for 24 h; after that, cells were stimulated with LPS (1 μg/mL) for 16 h. Then, Griess reagent was used to estimate the NO production in the medium. For the blank groups, we used a sterile culture medium. The quantity of nitrite in the samples was measured by using the sodium nitrite standard curve as a reference point.

### 2.8. Enzyme-Linked Immunosorbent Assay (ELISA) for Detecting TNF-α, IL-1β, IL-6, IL-8, INOS, COX-2, and IL-10 Production

ELISA assay for detecting TNF-α, IL-1β, IL-6, IL-8, IL-10, iNOS, and COX-2 production were pretreated with CCGA for 24 h and then induced with LPS (100 ng/mL) for 16 h. The culture supernatant was estimated using commercially available ELISA kits. All procedures were accomplished by following the manufactures’ instructions. The samples were analyzed in triplicate.

### 2.9. RNA Extraction and Real Time RT-PCR Analysis for Detecting TNF-α, IL-1β, IL-6, IL-8, IL-10, COX-2, and INOS Production

RAW264.7 cells were treated with different concentrations of CCGA for 24 h and stimulated with LPS. After 16 h, the supernatant was collected as the sample to be tested. Cells were blown with 1 mL Trizol and collected at −80 °C for RNA extraction. Afterward, RNA was extracted following the kit’s instructions. The qRT-PCR was performed with β-actin as the control. The primers used are presented in Table 1. The relative expression of the genes was deduced by the 2^−ΔΔ^ CT method, where, ΔCT = CT target gene—CT internal reference gene, ΔΔCt = ΔCT treated samples −ΔCT control sample, and multiple change = 2^−ΔΔ^ C.

### 2.10. Transient Transfection and Luciferase Activity Assay

The transient transfection and luciferase activity assay was carried out following the methods and procedures of Ying et al. [25].

### 2.11. Western Blot Analysis

Using a scraper, cells were collected after thorough washing in ice-cold phosphate-buffered saline (PBS); after that, a protein extraction kit (Beyotime Institute of Biotechnology, Jiangsu, China) was used to extract the cytoplasmic proteins. The protein was resolved using SDS-PAGE and transferred to PVDF membranes. The membranes were incubated with blocking buffer (5% (*w*/*v*) skimmed milk powder in 1TBS containing 0.1% Tween-20 for 1 h, then incubated overnight at 4 °C with a primary antibody (Santa Cruz Biotechnology, Santa Cruz, CA, USA). Membranes were incubated for 1 h at room temperature with HRP-linked secondary antibodies after the membrane was washed. Then, the membranes were washed again and detected using an enhanced chemiluminescence (ECL) kit and exposed to X-ray films (Kodak, Shanghai, China).

### 2.12. Statistical Analysis

Statistical analyses were performed with SPSS software, version 20.0 (SPSS, Inc., Chicago, IL, USA). All data are presented as means ± SD. One-way analysis of variance (ANOVA) followed by Tukey’s post hoc multiple comparison test was used and *p* < 0.01 was considered statistically significant.

## 3. Results

### 3.1. Detection of Cryptochlorogenic Acid by Thin Layer Chromatography

**Figure 2:**
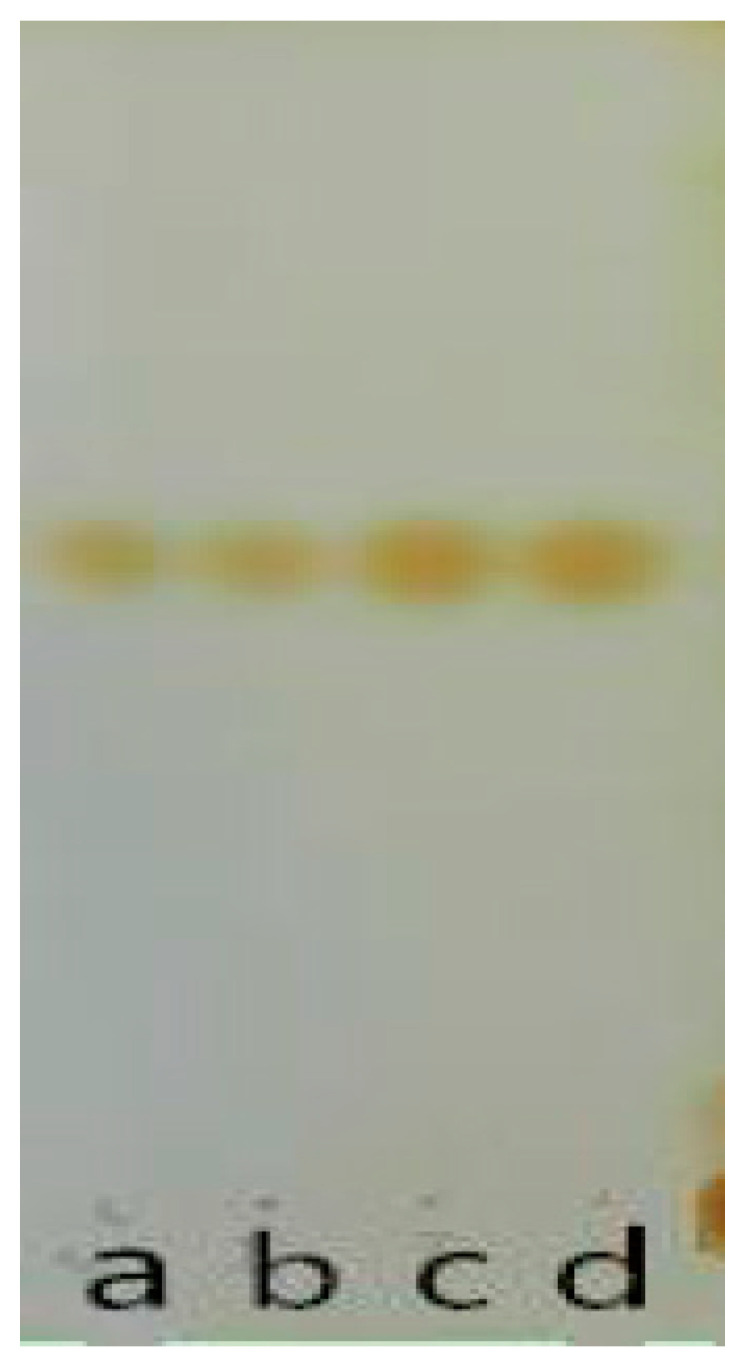
The results of TCL (a and b are standard substances; c and d are extractive monomers).

The results of the thin layer chromatography (TCL) are shown in Figure 2. The chromogenic points of the extracted monomers C and D in the thin layer chromatography are the same as those of A and B. Therefore, it was preliminarily inferred that the extracted monomers were cryptochlorogenic acid, and eluent with the same retention factors (RFs) was collected.

### 3.2. Determination of the Purity of Cryptochlorogenic Acid by Ultra High Performance Liquid Chromatography

#### 3.2.1. Standard Curve of Cryptochlorogenic Acid

**Figure 3:**
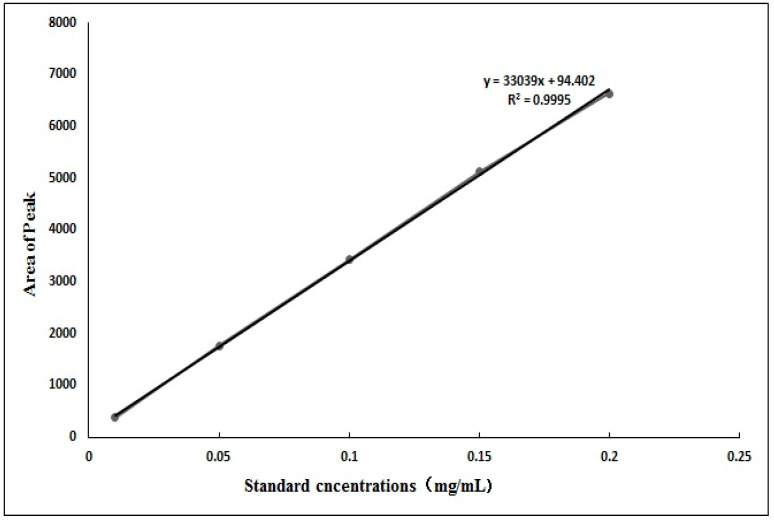
Standard linear regression curve.

As shown in Figure 3, according to UHPLC, the peak area of cryptochlorogenic acid standard was represented at the ordinate and the differential concentrations were represented at the abscissa. The graph showed the standard curve of cryptochlorogenic acid as y = 33039x + 94.402, where R*^2^* = 0.9995, indicating that there was a good linear relationship between cryptochlorogenic acid standard and its concentration.

#### 3.2.2. Determination of Cryptochlorogenic Acid in *Ageratina adenophora*

**Figure 4:**
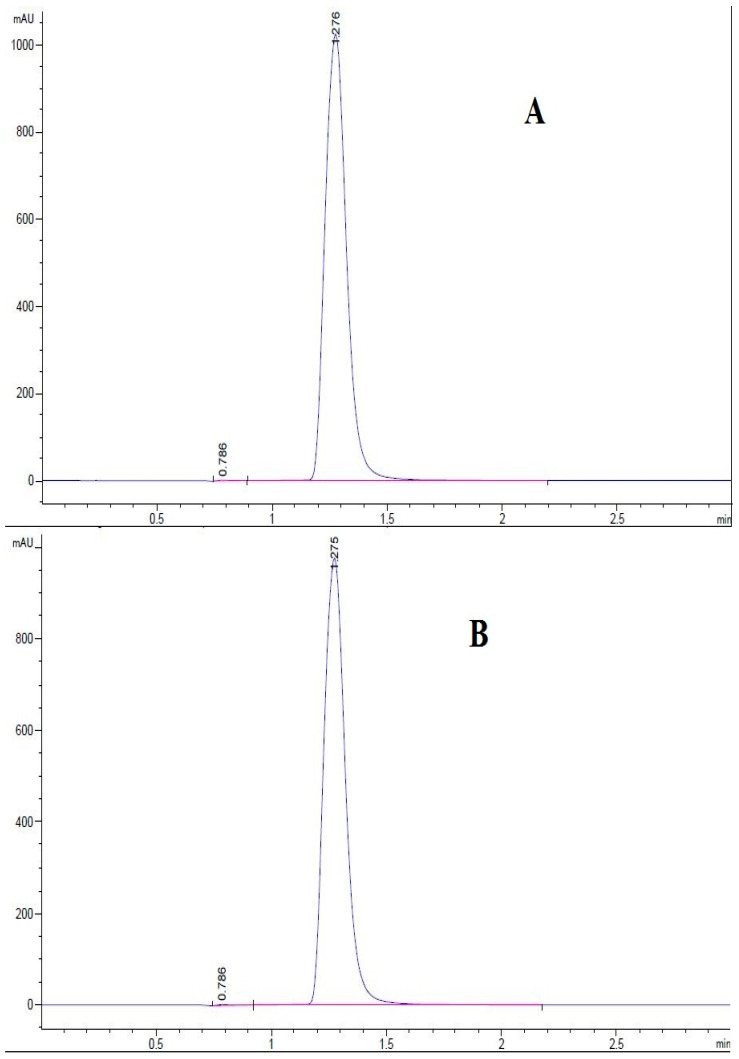
Determination of CCGA standard and extraction of CCGA from *A. adenophora* by UHPLC (**A**): Standard (0.2 mg/mL). (**B**): CCGA in *A. adenophora*.

The UHPLC determination results of the cryptochlorogenic acid standard and cryptochlorogenic acid extracted from *A. adenophora* at 325 nm are shown in Figure 4. The retention time of the cryptochlorogenic acid standard is 1.276 min, and the retention time of monomer extracted from *A. adenophora* was 1.275 min. It can be inferred that the monomer extracted from *A. adenophora* was cryptochlorogenic acid. In addition, the peak area of cryptochlorogenic acid extracted from *A. adenophora* in Figure 2 was 6324.19, i.e., y = 6324.19; the concentration of cryptochlorogenic acid after dissolution was 0.189 mg/mL; and the specific chlorogenic acid mass was 0.66 mg. It was calculated that the purity of cryptochlorogenic acid extracted from *A. adenophora* was 94.3%. Therefore, we concluded that the amount of CCGA in 0.7 mg extract was 0.6 mg and the purity was 94.3%.

### 3.3. Effects of CCGA on Cell Morphology and Cytotoxicity

**Figure 5:**
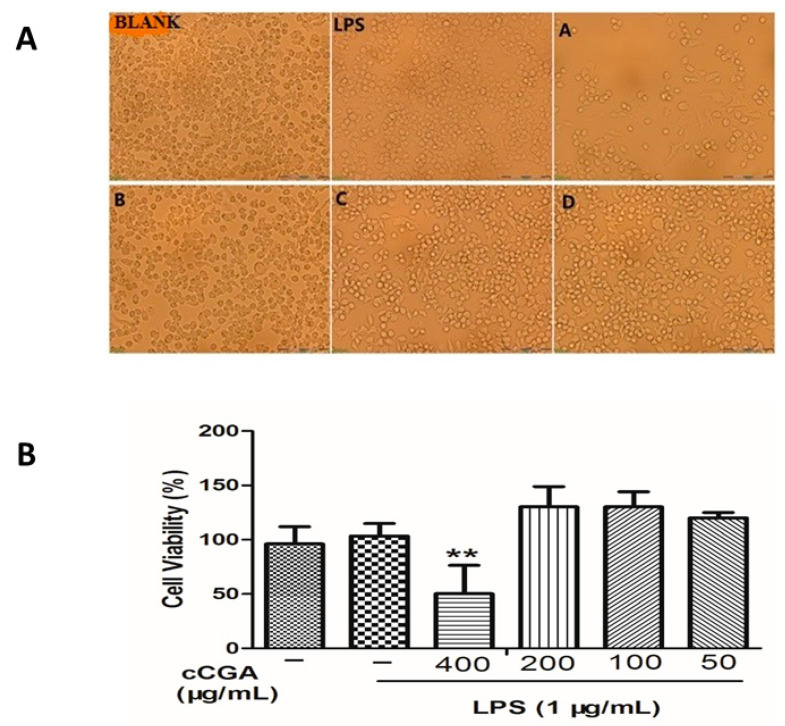
Cell morphology and viability after treatment with different concentrations of CCGA (BLANK: No LPS and CCGA treatments, LPS: Treatment with only LPS; A: 400 μg/mL CCGA + 1 μg/mL LPS; B: 200 μg/mL CCGA + 1 μg/mL LPS; C: 100 μg/mL CCGA + 1 μg/mL LPS; D: 50 μg/mL CCGA + 1 μg/mL LPS). (**A**) Photograph of viable cells after treatment with concentrations of CCGA (BLANK: No LPS and CCGA; LPS: Treatment with only LPS; A: 400 μg/mL CCGA + 1 μg/mL LPS; B: 200 μg/mL CCGA + 1 μg/mL LPS; C: 100 μg/mL CCGA + 1 μg/mL LPS; D: 50 μg/mL CCGA + 1 μg/mL LPS). (**B**) Viable cell count after treatment with concentrations of CCGA (A: 400 μg/mL; B: 200 μg/mL; C: 100 μg/mL; D: 50 μg/mL) Notes, ** *p* < 0.01 vs. LPS, *n* = 9 (mean ± standard deviation).

The experimental results (Figure 5A) showed that after CCGA treatment and LPS induction, the cells in the blank control, 200 μg/mL, 100 μg/mL, and 50 μg/mL groups were smooth in appearance without pseudopodia, and some cells were compact in distribution and aggregated; however, the cells in the LPS and 400 μg/mL group were elongated with pseudopodia, and most of them were spindle-shaped.

Furthermore, we observed that after 16 h, cells treated with CCGA at a concentration of 400 μg/mL were significantly lower than that of the LPS group (Figure 5B, *p* < 0.01), with a survival rate lower than 50%. Therefore, we concluded that CCGA at a concentration of 400 μg/mL was toxic to RAW264.7 cells. However, the 200, 100, and 50 μg/mL CCGA concentrations and the LPS groups had cell viability above 100%. Hence, we concluded that 1 μg/mL LPS and CCGA at concentrations of 200, 100, and 50 μg/mL did not show any obvious toxicity to RAW264.7 cells, and the maximum safe concentration of CCGA was 200 μg/mL. Therefore, CCGA concentrations of 200, 100, and 50 μg/mL were selected for subsequent tests.

### 3.4. Effect of CCGA on LPS-Induced Nitric Oxide (NO) and iNOS Production

**Figure 6:**
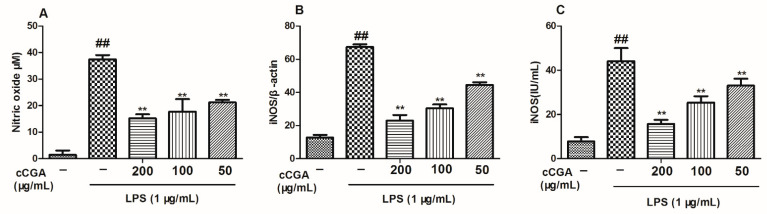
Effects of CCGA from *A. adenophora* on nitric oxide (NO) and iNOS production in LPS-induced RAW264.7 cells (**A**) Production level of NO after pretreatment with CCGA at 50–200 μg/mL for 24 h and then stimulation by LPS (1 μg/mL) for 16 h. (**B**) mRNA expression of iNOS after pretreatment with CCGA at 50–200 μg/mL for 24 h and then stimulation by LPS (1 μg/mL) for 16 h. (**C**) Protein levels of iNOS after pretreatment with CCGA at 50–200 μg/mL for 24 h and then stimulation by LPS (1 μg/mL) for 16 h. Notes: ## *p* < 0.01 vs. blank control group, ** *p* < 0.01 vs. LPS, *n* = 3 (mean ± standard deviation).

In this study, we observed that CCGA could reduce the production of NO and the expression of iNOS in LPS-induced RAW264.7 cells compared with the LPS group (Figure 6A−C, *p* < 0.01).

### 3.5. The Regulatory Effect of CCGA from A. adenophora on Inflammation Related Cytokines in LPS-Induced RAW264.7 Cells

**Figure 7:**
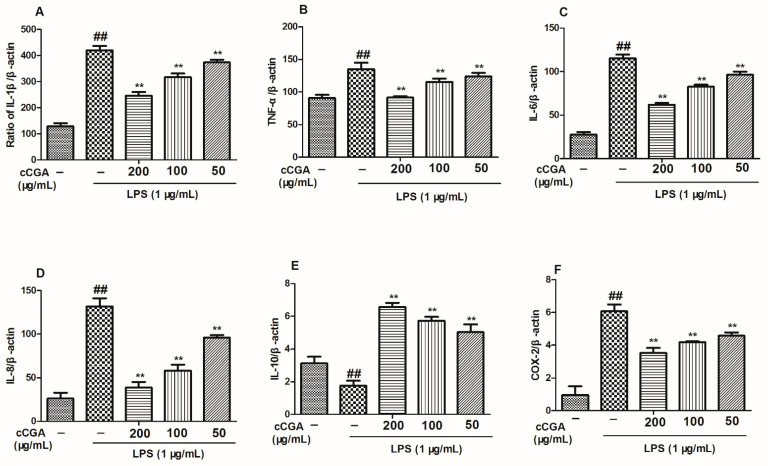
Effects of CCGA from *A. adenophora* on mRNA level of inflammatory-related cytokines in LPS-induced RAW264.7 cells. (**A**–**E**) mRNA level of inflammatory-related cytokines; (**F**) mRNA level of COX-2. Notes: ## *p* < 0.01 vs. blank control group, ** *p* < 0.01 vs. LPS, *n* = 3 (mean ± standard deviation).

**Figure 8:**
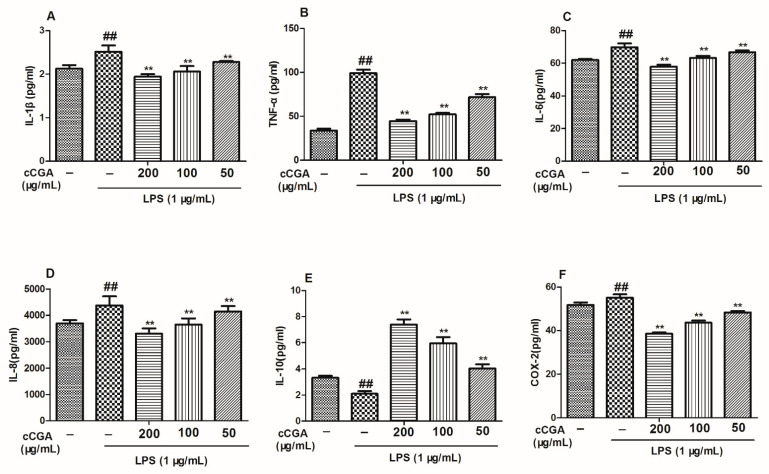
Effects of CCGA from *A. adenophora* on protein levels of inflammatory-related cytokines and COX-2 in LPS-induced RAW264.7 cells. (**A**–**E**) Protein levels of inflammatory-related cytokines. (**F**) Protein expression of COX-2. Notes: ## *p* < 0.01 vs. blank control group, ** *p* < 0.01 vs. LPS, *n* = 9 (mean ± standard deviation).

In this study, the mRNA and protein levels of pro-inflammatory cytokines (TNF- α, IL-1 β, IL-6, and IL-8) were elevated in the LPS group compared with the blank control group (Figure 7A–D and Figure 8A–D, *p* < 0.01). Furthermore, compared with the blank control group, anti-inflammatory cytokine IL-10 was downregulated in the LPS group (Figure 7E and Figure 8E, *p* < 0.01). In addition, the treatments groups reduced the expression levels of pro-inflammation cytokines (TNF- α, IL-1 β, IL-6, and IL-8) and increased the expression levels of IL-10 compared with the LPS group (Figure 7A–E and Figure 8A–E, *p* < 0.01).

### 3.6. Effect of CCGA from A. adenophora on COX-2 Expression

According to the results of this study, the mRNA and protein levels of COX-2 in the LPS group were higher than those in the control blank group (Figure 7F and Figure 8F, *p* < 0.01); however, CCGA could reduce the expression levels of COX-2 compared with the LPS group (Figure 5F and Figure 7F, *p* < 0.01).

### 3.7. CCGA Inhibited LPS-Induced NF-kB Activation in RAW 264.7 Cells

**Figure 9:**
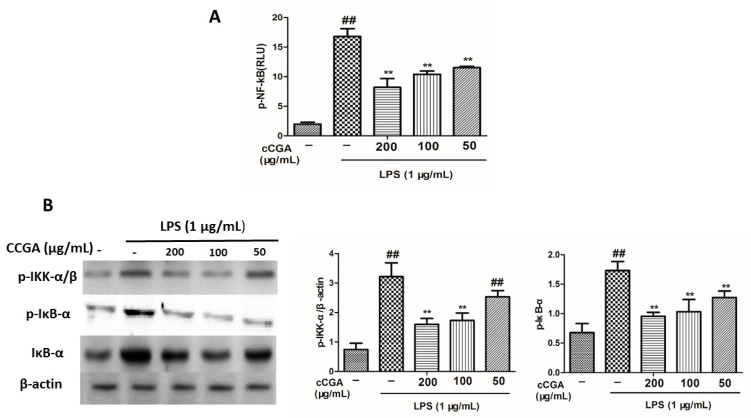
Effect of CCGA isolated from *A. adenophora* on phosphorylated proteins in NF-kB signaling pathway in LPS-induced RAW264.7 cells. (**A**) NF-kB transcriptional activity. Values are means ± standard deviation. (**B**) Protein levels of IkB-α, p-IkB-α, and p-IKK in RAW264.7 cells. Notes: ## *p* < 0.01 vs. blank control group, ** *p* < 0.01 vs. LPS, *n* = 9 (mean ± standard deviation).

To explore the anti-inflammatory mechanism of the CCGA, the NF-kB transcriptional activity was examined by the luciferase reporter gene assay. The results are represented in Figure 9A: CCGA reduced the LPS-induced NF-kB transcriptional activity in RAW 264.7 cells (*p* < 0.01). The main pathway known for the activation of NF-kB involves the phosphorylation of IKK and IκB, which activate the release and translocation of NF-kB into the nucleus. We further explored the cytoplasmic levels of p-IKK, IκB, and p-IκB using Western blot analysis to discover whether CCGA could regulate the NF-kB signaling pathway. We observed that CCGA prevented the phosphorylation of IKK and IκB and the degradation of the IκB protein after LPS stimulation (Figure 9B, *p* < 0.01).

## 4. Discussion

Plants contain secondary metabolites that are widely used in treating disease conditions [47]. These compounds are known to stimulate biochemical reactions in cells [48]. Pharmacological activities such as anti-aging, antioxidant, anti-cancer, and anti-inflammatory effects have been reported for these plant bioactive compounds [49]. Therefore, this study was conducted to ascertain the anti-inflammatory activity and mechanisms of cryptochlorogenic acid isolated from *A. adenophora,* an invasive and toxic plant. Our results demonstrate that CCGA at concentrations up to 200 μg/mL has better anti-inflammatory effects with no toxicity to cells; thus, the reduction in pro-inflammatory cytokine production was a result of CCGA activity and not cell death.

LPS activates inflammatory cytokines to terminate iNOS expression, thereby oxidizing L-arginine and increasing the production of NO [50]. NO, a free oxygen radical, is associated with the pathophysiology of several inflammatory diseases, as they act as cytotoxic agents in cells [51]. In activated macrophages, the production of NO may play a crucial role in the pathology of various acute and chronic inflammatory disorders [50]. Polyphenols decrease the production of NO in LPS induced inflammation. For example, a study by Moore et al. [52] reported that cranberry polyphenol could reduce the levels of NO in LPS-activated RAW 264.7 macrophages. Another study by Bigagli et al. [53] revealed that resveratrol and hydroxytyrosol synergistically inhibited the production of NO and PGE2 in LPS-stimulated RAW 264.7. In our study, we observed that LPS treatment elevated the NO and iNOS levels RAW264.7 cells; however, CCGA treatment could reduce NO production and downregulate the levels of iNOS in LPS-induced RAW264.7 cells. These results were similar to those of a study by Zhao et al. [37], which reported that CCGA at concentrations of 20, 40, and 80 μM reduced the production of NO and iNOS in LPS-induced RAW264.7 macrophages.

Numerous studies have reported a remarkable increase in pro-inflammatory cytokines in LPS-challenged macrophages and cells [54,55]. IL-1β, IL-6, and TNF-α are the most common cytokines associated with LPS-induced macrophages and have been reported to play vital roles in various biological functions, such as the control of the immune response, homeostasis, and inflammation [56]. In our study, the anti-inflammatory effect of CCGA on the mRNA and protein levels of inflammatory-related cytokines (TNF-α, IL-1β, IL-6, IL-8, COX-2, and IL-10) in LPS-induced RAW264.7 cells was elucidated. We observed that LPS treatment increased the production of inflammation-related cytokines (TNF- α, IL-1β, IL-6, IL-8, and COX-2) but reduced the levels of anti-inflammatory cytokine IL-10 in RAW264.7 cells. However, CCGA could prevent the production of these inflammatory cytokines (TNF- α, IL-1β, IL-6, IL-8, and COX-2) both at the mRNA and protein levels, whereas it enhanced the production of anti-inflammatory cytokines (IL-10) in RAW264.7 cells. These results are consistent with the study by Zhao et al. [37], which reported that the production of pro-inflammatory cytokines (TNF-α, IL-6, and COX-2) was reduced after treatment with 150 μM CCGA in LPS-induced inflammation in RAW264.7 macrophages. Similarly, Kim et al. [57] also reported that chlorogenic acid could suppress lipopolysaccharide-induced nitric oxide and interleukin-1β expression by hindering JAK2/STAT3 activation in RAW264.7 cells. Therefore, we conclude that CCGA prevented LPS-induced inflammation in RAW264.7 cells by inhibiting the production of inflammation-inducing cytokines.

NF-κB is the key tool for the regulation of pro-inflammatory enzymes and cytokines [58]. The initiation of the NF-κB pathway is regulated by IκB-α, whereas IκB kinase (IKK) regulates the degradation of IκB-α. [59]. Therefore, when IKK phosphorylates, it results in the degradation of IκB-α, which then activates NF-κB p65 to stimulate the production of pro-inflammatory factors in the cytoplasm [60,61]; hence, the suppression of the NF-κB signaling pathway is a promising strategy for the treatment of inflammatory disorders. Numerous studies have revealed several anti-inflammatory polyphenols that suppress NF-κB signaling [62]. Yang et al. [63] revealed that resveratrol inhibited the initiation of the NF-κB signaling pathway. In addition, another study by Serreli and Deiana [64] also reported that extra virgin olive oil polyphenols could inhibit NF-κB activation, thus reducing the levels of pro-inflammatory factors. Therefore, we investigated the effects of CCGA on the total phosphorylation of NF-κB family proteins (IKK and IκB) in RAW264.7 cells induced by LPS using Western blot. Our results showed that pretreatment with CCGA significantly blocked NF-kB promoter luciferase activity and downregulated the phosphorylation levels of IKK and IκB in LPS-induced RAW264.7 cells in a dose-dependent manner. Similar results were recorded in the study of Li et al. [65]. Therefore, we concluded that CCGA isolated from *A. adenophora* prevented inflammation by inhibiting the phosphorylation of NF-κB proteins. However, the exact molecular mechanism of the NF-κB signaling pathway inhibition by CCGA is not clear and thus requires further studies.

## 5. Conclusions

In conclusion, CCGA treatment reduced the levels of NO, iNOS, COX-2, and pro-inflammation cytokines in LPS-induced RAW264.7 cells. The anti-inflammatory properties of CCGA are achieved by blocking or suppressing the NF-κB signaling pathway in RAW 264.7 cells. Therefore, CCGA isolated from *Ageratina adenophora* can be used as a potential therapeutic agent for the treatment of inflammation-related diseases. However, there is a need for further studies on the exact activity of CCGA in inhibiting the NF-κB inflammation signaling pathway.

## Figures and Tables

**Table 1:** Primers used for qRT-PCR.

Genes	Primer Sequence (5′→3′)	References
TNF-α	F- AGCACAGAAAGCATGATCCG R- ATGAGAGGGAGGCCATT	[10]
IL-1β	F- GTTCCCCAACTGGTACATCA R- CCATACTTTAGGAAGACACGG	[11]
IL-6	F- CTTCTTGGGACTGATGCTGGT G R- CGCTGGCTTTGTCTTTCTTGTTA	[12]
IL-8	F- CAAGGCTGGTCCATGCTCC R- TGCTATCACTTCCTTTCTGTTG	[11]
IL-10	F- GCTCTTACTGACTGGCATGAG R- CGCAGCTCTAGGAGCATGTG	[13]
INOS	F- TCCTACACCACACCAAAC R- CTCCAATCTCTGCCTATCC	[10]
COX	F- TGCTGTACAAGCAGTGGCAA R- GCAGCCATTTCCTTCTCTCC	[10]
β-actin	F- CGGTTGGCCTTAGGGTTCAGGGGGG R- GTGGGCCGCTCTAGGCACCA	[12]

F, forward; R, reverse.

## Data Availability

The data presented in this study are available on request from the corresponding author.
